# Chemistry for Dental Students

**Published:** 1878-04

**Authors:** 


*Chemistry.*—This noble, experimental and exact science, must in some future time confer a great blessing on the dental profession.

It is through experimental Chemistry, that the dental cement is to be discovered, which will take the place of nearly all these *stoppings*. I hope the discovery will be made by a practical dentist. Chemistry is a fascinating study, and it is a pity that so few dental students are aware of it. Fifty dollars worth of chemical apparatus in the laboratory, would give amusement and instruction to many young practitioners, who have some leisure in their early professional life. Vast good would ultimately arise from even a *small* army of *experimenters*.

